# The 100 Most Influential Studies in Chimeric Antigen Receptor T-Cell: A Bibliometric Analysis

**DOI:** 10.3389/fmedt.2020.00003

**Published:** 2020-09-22

**Authors:** Beomjun Seo, Seungwook Kim, Jeeyoon Kim

**Affiliations:** ^1^Department of Epidemiology and Medical Informatics, Graduate School of Public Health, Korea University, Seoul, South Korea; ^2^Graduate School of Interdisciplinary Management, Ulsan National Institute of Science and Technology (UNIST), Ulsan, South Korea; ^3^Department of Clinical Pharmacy, Graduate School, Cha University, Seoul, South Korea

**Keywords:** chimeric antigen receptors, chimeric antigen receptor T-cell, citation classic, bibliometric, Web of Science, VOSviewer, CAR-T

## Abstract

**Background:** Bibliometric analyses are used to provide information on trends within a specific research field, along with indicators of the impact of a publication. With such an analysis, we map the scientific landscape of chimeric antigen receptor T-cell (CAR-T) research to see the emerging topics and infer directions the field might take.

**Methods:** We extracted the 100 most-cited articles, published all periods (from 2008 to 2019) by the Web of Science Core Collection. Using their bibliographic details, including year of publication, country of author, research organization, author information, and keywords, we graph the networks created between the articles.

**Results:** Of the 100 papers identified, the majority (93%) were written in the USA. Notable was that 34 papers were published from the University of Pennsylvania. Regarding authors, Carl H. June participated in 29 researches, followed by Bruce L. Levine who participated in 12. As for journals, *Blood* (*n* = 19) published the most papers, followed by *Science Translational Medicine* (*n* = 9) and *Cancer Research* (*n* = 9). Lastly, the most frequently used keywords were “adoptive immunotherapy” (*n* = 47), “lymphocytes” (*n* = 27), and “antitumor activity” (*n* = 22).

**Conclusion:** By evaluating the top 100 most-cited papers in the CAR-T field, this study provides insight into the direction of the scientific growth and its trends, as well as information on the field's network structure.

## Introduction

Since chimeric antigen receptors (CARs) were genetically engineered to express on T-cells three decades ago (1), they have become one of the most promising targeted immunotherapy research interests. These CAR T-cells (CAR-Ts) are modified to express cancer antigen-recognizing CARs and to stimulate the immune system (2–4). To produce CAR-Ts, leukapheresis is performed in the patient's blood: first, the T-cell is extracted (selection and activation phase) with the virus vector (retrovirus/lentivirus vector), and then the host T-cell is injected with the unique cancer-specific CAR DNA (CAR transduction) for cell proliferation (expansion). It is critical that these methods, such as the handling of the virus and the quality of the CAR-T, are properly verified as these manufactured CAR-Ts are reinfused into the patient, and any errors in selection may compromise their health. The success of these early researches and trials sets the basis for a larger clinical trial in a CD19-targeted CAR-T therapy called tisagenlecleucel (Kymriah™) for children and adolescents with acute lymphoblastic leukemia. Based on the clinical trial results, the Food and Drug Administration (FDA) approved tisagenlecleucel as the first treatment in August 2017. Currently, only two multinational pharmaceutical companies (Novartis and Gilead) have FDA approval for the treatment of some hematologic disorder (Table 1).

**Table 1:** Status of chimeric antigen receptor T-cell (CAR-T) approved by the Food and Drug Administration (FDA) or in Phase III clinical trials (as of July 1, 2019).

	**Kymriah^®^ (tisagenlecleucel-T; CTL019)**	**Yescarta^®^ (axicabtagene ciloleucel; KTE-C19)**	**Lisocabtagene maraleucel (liso-cel, JCAR-017)**	**BB2121**
Company	Novartis	Gilead (Kite)	Celgene	Celgene/Bluebird
Format	CAR^a^-T	CAR-T	CAR-T	CAR-T
Co-stimulation	4-1BB CS	CD^f^28 CS	4-1BB CS	4-1BB CS
Phase III name	B-ALL^b^: ELINA/DLBCL^c^: JULIET	TOWER	TRANSFORM	RRMM
Status	Approved by USFDA	Approved by USFDA	Phase III (NCT03575351)	Phase III (NCT03651128)
Cost (US)	$475,000 for B-ALL; $373,000 for R/R^d^ DLBCL	$373,000	NA^h^	NA
Indication	B-ALL, R/R DLBCL	R/R DLBCL; PMBCL^g^	R/R DLBCL; CLL^i^	MM^j^
Sourced T-cell	Patient PBMCs^e^; autologous; unspecified	Patient PBMCs; autologous; unspecified	Patient CD4 and CD8 T cells 1:1 ratio; autologous	Patient PBMCs; autologous
Vector	Lentivirus	Retrovirus	Lentivirus	Lentivirus
Patient group (=no.)	B-ALL = 63, R/R DLBCL = 93	101	73^*^	33^*^
ORR	B-ALL = ND, DLBCL = 52%	83%	80%^*^	85%^*^
CR	B-ALL = 83%, DLBCL = 40%	58%	59%^*^	45%^*^
PR	B-ALL = 20%, DLBCL = 12%	25%	21%^*^	39%
Median response duration time (months)	B-ALL = NR, DLBCL = 11.7 months	11.1 months	10.2 months^*^	11.8 months^*^

a *CAR, chimeric antigen receptor*.

b *B-ALL, B-cell acute lymphoblastic leukemia*.

c *DLBCL, diffuse large B-cell lymphoma*.

d *R/R, relapsed or refractory*.

e *PBMC, peripheral blood mononuclear cells*.

f *CD, cluster of differentiation*.

g *PMBCL, primary mediastinal large B-cell lymphoma*.

h *NA, not applicable*.

i *CLL, chronic lymphocytic leukemia*.

j *MM, multiple myeloma*.

* *Data from the Phase I trial result*.

The CAR-T studies registered at ClinicalTrials.gov as ongoing (i.e., not yet recruiting, recruiting, enrolling by invitation, and active but not recruiting) number over 300. Among them are studies that look into the development of new combination therapeutic options for malignant blood cancer, but also solid tumors, and other immunotherapies (5–7). In many therapeutic areas, immunotherapy using CD19-targeted CAR-T therapy is being introduced as a new and promising treatment for systemic lupus erythematosus (SLE), regulatory rheumatoid factor (regRF) bring into being lymphocytes also use therapeutic targets in rheumatoid arthritis (8–10).

Not only has the number of CAR-T experiments been growing, but over 3,000 articles regarding CAR-Ts have been published according to Clarivate Analytics' Web of Science Core Collection (WoSCC; www.webofknowledge.com). The WoSCC online database provides systematic literature information, including data under the scope of the Science Citation Index (SCI) and the Science Citation Index Expanded (SCIE), Social Science Citation Index (SSCI), Arts and Humanity Citation Index (A&HCI), and the Emerging Sources Citation Index (ESCI) (11).

Typically, citations express an author's consent to a study's presented insights, findings, and interpretations presented. Thus, citation analysis, as a quantitative bibliometric method, can be used to provide information on a study's trends, as well as an objective index of the scientific effect of publications through their citation frequency within a specific field (9–11). Consequently, the most-cited articles are analyzed through their bibliometrics to understand the direction of scientific growth and flow of the study area (12–14).

In this study, a bibliometric network analysis will be conducted on the 100 most-cited publications in the CAR-T area. Using their bibliographic information (i.e., year of publication, country, funding, institution, author information, and keywords), we can provide insights on the target cells and genes typically studied in the field, indications for major studies, and any hot topics regarding CAR-Ts after analyzing the simultaneous exposure and frequency of the core keywords. Also, the information yielded may allow us to determine potential new study fields, the opportunities available to researchers, and leading funding organizations.

## Methods

Using “chimeric antigen receptor T cell,” “CAR-T,” or “CAR T” as keywords, we found a total of 3,871 articles published from January 1, 2008, to December 31, 2019. All of them were located using the WoSCC as of May 8, 2020. Based on the target period, the top 100 cited articles were reviewed, and there were excellent techniques and quantitative growth such as immuno-oncology and gene therapy area. The most highly cited papers are listed in Table 2.

**Table 2:** The 100 most-cited articles from the *Journal of CAR-T*.

**Title**	**Journal**	**Published year**	**Total citation (*n*)**
T cells with chimeric antigen receptors have potent antitumor effects and can establish memory in patients with advanced leukemia	*Science Translational Medicine*	2011	1,189
T cells expressing CD19 chimeric antigen receptors for acute lymphoblastic leukemia in children and young adults: a phase 1 dose-escalation trial	*Lancet*	2015	1,175
Efficacy and toxicity management of 19-28z CAR T cell therapy in B cell acute lymphoblastic leukemia	*Science Translational Medicine*	2014	1,102
Chemotherapy-refractory diffuse large B-cell lymphoma and indolent B-cell malignancies can be effectively treated with autologous T cells expressing an Anti-CD19 chimeric antigen receptor	*Journal of Clinical Oncology*	2015	772
Axicabtagene ciloleucel CAR T-cell therapy in refractory large B-cell lymphoma	*New England Journal of Medicine*	2017	728
Tisagenlecleucel in children and young adults with B-cell lymphoblastic leukemia	*New England Journal of Medicine*	2018	722
Chimeric antigen receptor T cells persist and induce sustained remissions in relapsed refractory chronic lymphocytic leukemia	*Science Translational Medicine*	2015	629
CD19 CAR-T cells of defined CD4(+): CD8(+) composition in adult B cell ALL patients	*Journal of Clinical Investigation*	2016	579
CD28 costimulation improves expansion and persistence of chimeric antigen receptor-modified T cells in lymphoma patients	*Journal of Clinical Investigation*	2011	568
Antitumor activity and long-term fate of chimeric antigen receptor-positive T cells in patients with neuroblastoma	*Blood*	2011	536
Long-term follow-up of CD19 CAR therapy in acute lymphoblastic leukemia	*New England Journal of Medicine*	2018	426
4-1BB costimulation ameliorates T cell exhaustion induced by tonic signaling of chimeric antigen receptors	*Nature Medicine*	2015	406
Regression of glioblastoma after chimeric antigen receptor T-cell therapy	*New England Journal of Medicine*	2016	398
CAR T cell immunotherapy for human cancer	*Science*	2018	373
Targeting a CAR to the TRAC locus with CRISPR/Cas9 enhances tumor rejection	*Nature*	2017	372
Mesothelin-specific chimeric antigen receptor mRNA-engineered T cells induce antitumor activity in solid malignancies	*Cancer Immunology Research*	2014	372
Antibody-modified T cells: CARs take the front seat for hematologic malignancies	*Blood*	2014	363
Human epidermal growth factor receptor 2 (HER2)-specific chimeric antigen receptor-modified T cells for the immunotherapy of HER2-positive sarcoma	*Journal of Clinical Oncology*	2015	358
The future of cancer treatment: immunomodulation, CARs and combination immunotherapy	*Nature Reviews Clinical Oncology*	2016	351
Chimeric antigen receptor T-cell therapy—assessment and management of toxicities	*Nature Reviews Clinical Oncology*	2018	349
Immunotherapy of non-Hodgkin's lymphoma with a defined ratio of CD8(+) and CD4(+) CD19-specific chimeric antigen receptor-modified T cells	*Science Translational Medicine*	2016	338
Toxicities of chimeric antigen receptor T cells: recognition and management	*Blood*	2016	327
Anti-PD-1 antibody therapy potently enhances the eradication of established tumors by gene-modified T cells	*Clinical Cancer Research*	2013	323
Treatment of metastatic renal cell carcinoma with CAIX CAR-engineered T cells: clinical evaluation and management of on-target toxicity	*Molecular Therapy*	2013	308
Decade-long safety and function of retroviral-modified chimeric antigen receptor T cells	*Science Translational Medicine*	2012	308
Donor-derived CD19-targeted T cells cause regression of malignancy persisting after allogeneic hematopoietic stem cell transplantation	*Blood*	2013	304
CD19-targeted chimeric antigen receptor T-cell therapy for acute lymphoblastic leukemia	*Blood*	2015	299
The principles of engineering immune cells to treat cancer	*Cell*	2017	296
Tumor-targeted T cells modified to secrete IL-12 eradicate systemic tumors without need for prior conditioning	*Blood*	2012	296
Molecular remission of infant B-ALL after infusion of universal TALEN gene-edited CAR T cells	*Science Translational Medicine*	2017	288
A single dose of peripherally infused EGFRvIII-directed CAR T cells mediates antigen loss and induces adaptive resistance in patients with recurrent glioblastoma	*Science Translational Medicine*	2017	280
CD22-targeted CAR T cells induce remission in B-ALL that is naive or resistant to CD19-targeted CAR immunotherapy	*Nature Medicine*	2018	270
Chimeric antigen receptor therapy	*New England Journal of Medicine*	2018	264
Identification of predictive biomarkers for cytokine release syndrome after chimeric antigen receptor T-cell therapy for acute lymphoblastic leukemia	*Cancer Discovery*	2016	256
T cells expressing an anti-B-cell maturation antigen chimeric antigen receptor cause remissions of multiple myeloma	*Blood*	2016	246
Toxicity and management in CAR T-cell therapy	*Molecular Therapy—Oncolytics*	2016	246
T cells expressing chimeric antigen receptors can cause anaphylaxis in humans	*Cancer Immunology Research*	2013	246
Human CAR T cells with cell-intrinsic PD-1 checkpoint blockade resist tumor-mediated inhibition	*Journal of Clinical Investigation*	2016	241
Tisagenlecleucel in adult relapsed or refractory diffuse large B-cell lymphoma	*New England Journal of Medicine*	2019	235
Multiple injections of electroporated autologous T cells expressing a chimeric antigen receptor mediate regression of human disseminated tumor	*Cancer Research*	2010	235
Distinct signaling of coreceptors regulates specific metabolism pathways and impacts memory development in CAR T cells	*Immunity*	2016	234
Treating B-cell cancer with T cells expressing anti-CD19 chimeric antigen receptors	*Nature Reviews Clinical Oncology*	2013	233
Endothelial activation and blood-brain barrier disruption in neurotoxicity after adoptive immunotherapy with CD19 CAR-T cells	*Cancer Discovery*	2017	229
Chimeric antigen receptor-modified T cells derived from defined CD8(+) and CD4(+) subsets confer superior antitumor reactivity *in vivo*	*Leukemia*	2016	227
Intent-to-treat leukemia remission by CD19 CAR T cells of defined formulation and dose in children and young adults	*Blood*	2017	222
Allogeneic t cells that express an anti-CD19 chimeric antigen receptor induce remissions of B-cell malignancies that progress after allogeneic hematopoietic stem-cell transplantation without causing graft-vs.-host disease	*Journal of Clinical Oncology*	2016	217
Multiplex genome editing to generate universal CAR T cells resistant to PD1 inhibition	*Clinical Cancer Research*	2017	216
Structural design of engineered costimulation determines tumor rejection kinetics and persistence of CAR T cells	*Cancer Cell*	2015	216
Expression of a functional CCR2 receptor enhances tumor localization and tumor eradication by retargeted human T cells expressing a mesothelin-specific chimeric antibody receptor	*Clinical Cancer Research*	2011	216
Design and development of therapies using chimeric antigen receptor-expressing T cells	*Immunological Reviews*	2014	213
Driving CAR T-cells forward	*Nature Reviews Clinical Oncology*	2016	208
Acquisition of a CD19-negative myeloid phenotype allows immune escape of MLL-rearranged B-ALL from CD19 CAR-T-cell therapy	*Blood*	2016	207
A foundation for universal T-cell based immunotherapy: T cells engineered to express a CD19-specific chimeric-antigen-receptor and eliminate expression of endogenous TCR	*Blood*	2012	206
Receptor affinity and extracellular domain modifications affect tumor recognition by ROR1-specific chimeric antigen receptor T cells	*Clinical Cancer Research*	2013	203
Redirecting T-cell specificity by introducing a tumor-specific chimeric antigen receptor	*Blood*	2010	197
Phase 1 results of ZUMA-1: a multicenter study of KTE-C19 anti-CD19 CAR T cell therapy in refractory aggressive lymphoma	*Molecular Therapy*	2017	196
Rational development and characterization of humanized anti-EGFR variant III chimeric antigen receptor T cells for glioblastoma	*Science Translational Medicine*	2015	193
Closely related T-memory stem cells correlate with *in vivo* expansion of CAR.CD19-T cells and are preserved by IL-7 and IL-15	*Blood*	2014	190
Persistence and efficacy of second generation CAR T cell against the LeY antigen in acute myeloid leukemia	*Molecular Therapy*	2013	188
Regional delivery of mesothelin-targeted CAR T cell therapy generates potent and long-lasting CD4-dependent tumor immunity	*Science Translational Medicine*	2014	187
Heparanase promotes tumor infiltration and antitumor activity of CAR-redirected T lymphocytes	*Nature Medicine*	2015	183
Going viral: chimeric antigen receptor T-cell therapy for hematological malignancies	*Immunological Reviews*	2015	181
Immune responses to transgene and retroviral vector in patients treated with *ex vivo*-engineered T cells	*Blood*	2011	181
Cancer immunotherapy: harnessing the immune system to battle cancer	*Journal of Clinical Investigation*	2015	180
Monocyte-derived IL-1 and IL-6 are differentially required for cytokine-release syndrome and neurotoxicity due to CAR T cells	*Nature Medicine*	2018	179
Determinants of response and resistance to CD19 chimeric antigen receptor (CAR) T cell therapy of chronic lymphocytic leukemia	*Nature Medicine*	2018	177
T cells expressing CD123-specific chimeric antigen receptors exhibit specific cytolytic effector functions and antitumor effects against human acute myeloid leukemia	*Blood*	2013	177
The nonsignaling extracellular spacer domain of chimeric antigen receptors is decisive for *in vivo* antitumor activity	*Cancer Immunology Research*	2015	175
Kinetics and biomarkers of severe cytokine release syndrome after CD19 chimeric antigen receptor-modified T-cell therapy	*Blood*	2017	174
Phase I trials using Sleeping Beauty to generate CD19-specific CAR T cells	*Journal of Clinical Investigation*	2016	174
Multiplex genome-edited T-cell Manufacturing platform for “off-the-shelf” adoptive T-cell immunotherapies	*Cancer Research*	2015	174
Tandem CAR T cells targeting HER2 and IL13R alpha 2 mitigate tumor antigen escape	*Journal of Clinical Investigation*	2016	170
IL-12 release by engineered T cells expressing chimeric antigen receptors can effectively muster an antigen-independent macrophage response on tumor cells that have shut down tumor antigen expression	*Cancer Research*	2011	170
Redirecting specificity of T-cell populations for CD19 using the Sleeping beauty system	*Cancer Research*	2008	169
Durable Molecular remissions in chronic lymphocytic leukemia treated with CD19-specific chimeric antigen receptor-modified T cells after failure of ibrutinib	*Journal of Clinical Oncology*	2017	164
Engineered CAR T cells targeting the cancer-associated Tn-glycoform of the membrane mucin MUC1 control adenocarcinoma	*Immunity*	2016	164
T cells expressing CD19/CD20 bispecific chimeric antigen receptors prevent antigen escape by malignant B cells	*Cancer Immunology Research*	2016	162
*In vivo* persistence, tumor localization, and antitumor activity of CAR-engineered T cells is enhanced by costimulatory signaling through CD137 (4-1BB)	*Cancer Research*	2011	161
Multifactorial T-cell hypofunction that is reversible can limit the efficacy of chimeric antigen receptor-transduced human T cells in solid tumors	*Clinical Cancer Research*	2014	159
Recognition of glioma stem cells by genetically modified T cells targeting EGFRvIII and development of adoptive cell therapy for glioma	*Human Gene Therapy*	2012	157
CAR T cell-induced cytokine release syndrome is mediated by macrophages and abated by IL-1 blockade	*Nature Medicine*	2018	155
A chimeric switch-receptor targeting PD1 augments the efficacy of second-generation CAR T cells in advanced solid tumors	*Cancer Research*	2016	151
CD27 co-stimulation augments the survival and antitumor activity of redirected human T cells *in vivo*	*Blood*	2012	151
Affinity-tuned ErbB2 or EGFR chimeric antigen receptor T cells exhibit an increased therapeutic index against tumors in mice	*Cancer Research*	2015	149
Targeting fibroblast activation protein in tumor stroma with chimeric antigen receptor T cells can inhibit tumor growth and augment host immunity without severe toxicity	*Cancer Immunology Research*	2014	147
CD19-targeted CAR T-cell therapeutics for hematologic malignancies: interpreting clinical outcomes to date	*Blood*	2016	145
CAR T cell therapy for solid tumors	NA	2017	144
Ibrutinib enhances chimeric antigen receptor T-cell engraftment and efficacy in leukemia	*Blood*	2016	142
Chimeric antigen receptor T cells with dissociated signaling domains exhibit focused antitumor activity with reduced potential for toxicity *in vivo*	*Cancer Immunology Research*	2013	142
Long-term safety and activity of axicabtagene ciloleucel in refractory large B-cell lymphoma (ZUMA-1): a single-arm, multicentre, phase 1-2 trial	*Lancet Oncology*	2019	141
Tuning sensitivity of CAR to EGFR density limits recognition of normal tissue while maintaining potent antitumor activity	*Cancer Research*	2015	136
Tumor-promoting desmoplasia is disrupted by depleting FAP-expressing stromal cells	*Cancer Research*	2015	134
Global manufacturing of CAR T cell therapy	*Molecular Therapy-Methods & Clinical Development*	2017	133
Mesothelin-targeted CARs: driving T cells to solid tumors	*Cancer Discovery*	2016	133
Novel immunotherapies in lymphoid malignancies	*Nature Reviews Clinical Oncology*	2016	131
Phase I hepatic immunotherapy for metastases study of intra-arterial chimeric antigen receptor-modified T-cell therapy for CEA(+) liver metastases	*Clinical Cancer Research*	2015	131
Chimeric antigen receptor T-cell therapies for lymphoma	*Nature Reviews Clinical Oncology*	2018	130
Switch-mediated activation and retargeting of CAR-T cells for B-cell malignancies	*Proceedings of the National Academy of Sciences of the United States of America*	2016	129
CRISPR/Cas9-mediated PD-1 disruption enhances anti-tumor efficacy of human chimeric antigen receptor T cells	*Scientific Reports*	2017	128
ICOS-based chimeric antigen receptors program bipolar T(H)17/T(H)1 cells	*Blood*	2014	127

All publication information (i.e., journal name, country, enhanced-organization information, author, title, publisher, keyword, PubMed ID and citation frequency, and references) was downloaded as Bib-Text files and then converted into the XML format. The network graph was constructed using VOSviewer 1.6.13, a software tool for visualizing and exploring network data. The VOSviewer manual by Van Eck and Waltman states that VOSviewer is a program for building and visualizing networks of scientific publications, journals, researchers, research institutions, countries, keywords, or terms. It can be used to analyze bibliographic and other types of networks (15). In this study, we used the function of the VOSviewer 1.6.13 version to design the network graph. For clearer network visualization, keywords can be marked more than five times and author networks more than two times. For brevity of presentation, only information on the top 10 is included in the tables. This noninterference study conducted a bibliometric analysis not involving any human subjects; thus, no approval was required from any institutional review board or ethics committee.

## Result

**Figure 1:**
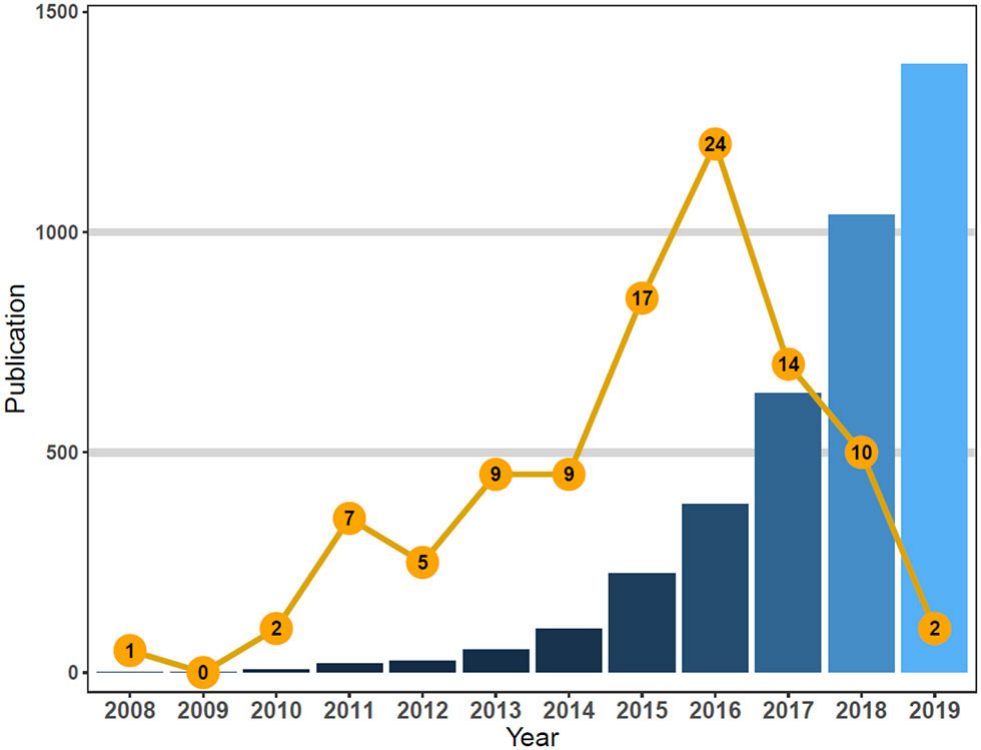
Total chimeric antigen receptor T-cell (CAR-T)-related publications vs. top-cited publications from 2008 to 2019.

The publication rate and the citation frequency of the 100 most-cited publications are shown in Figure 1.

The total citation frequencies of the articles ranged from 1,189 to 127 (μ = 277; Md = 213). The top 30% (>34 rank) were referenced an average of 468.3 times. Of the 100 articles published across the decade, 58% were published in 2015 (*n* = 17), 2016 (*n* = 24), and 2017 (*n* = 14), with the most referenced publications found in 2016 (Figure 1).

We observed that majority of the publications were original articles (80%), while the remaining were reviews (20%); all publications were released in English across 28 different journals. The top 100 most-cited articles were published by co-authors from 19 countries—most of who were from the USA (*n* = 93), Germany (*n* = 11), Italy (*n* = 6), the UK (*n* = 5), and Canada (*n* = 5) (Table 3).

**Table 3:** Status of countries/funding organizations that top 100 cited publications related to chimeric antigen receptor T-cell (CAR-T).

**Rank**	**Country**	**Number of publications**	**ACPI^**a**^**	**ACPY^**b**^**	**Sum of times cited**
1	USA	93	286	2,046	26,598
2	Germany	11	320	352	3,520
3	Italy	6	275	165	1,653
4	Canada	5	435	311	2,179
5	Australia	4	177	148	888
6	England	4	377	188	1,511
7	Australia	3	390	195	1,170
8	France	3	148	44	446
9	China	2	496	330	992
10	Israel	2	439	175	879

a *ACPI, average citations per item (average number of times a record has been cited) (11)*.

b *ACPY, average citations per year (average number of times a record has been cited) (11)*.

**Figure 2:**
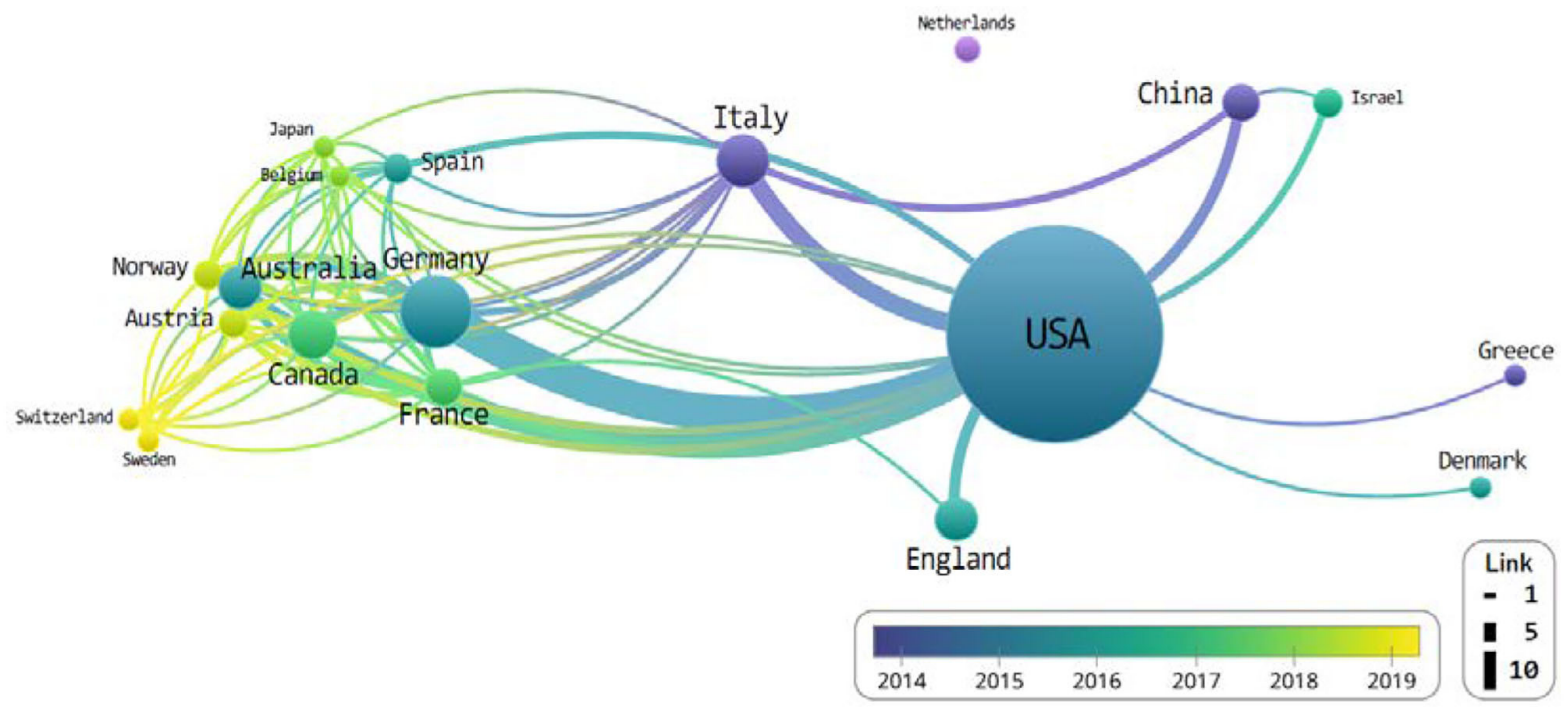
The network of countries that co-published related to chimeric antigen receptor T-cell (CAR-T) from 2018 to June 2019.

The results are able to note that the USA and Germany showed the most pronounced activity (Figure 2). The thickness of the link is expressed according to the number of connected nodes.

Hartmann et al. (16) corroborates this lead the USA possesses as he cites their overwhelming percentage of next-generation development and research in the rapidly developing field of CAR-T, similar to their development of new technologies and cost-intensive immunotherapies.

The articles were published across 28 journals, focusing on subjects such as blood cancer, chemotherapy research, and immune-chemotherapies; all of them were located in the USA (Table 3). Notably, it was the journals *Blood* (*n* = 19), *Cancer Research* (*n* = 9), and *Science Translational Medicine* (*n* = 9) that accounted for 37% of the publications, 14 of which had only published one paper (Table 4).

**Table 4:** Status of journal titles that top 100 cited publications related to chimeric antigen receptor T-cell (CAR-T).

**Rank**	**Journal titles**	**Number of publications**	**APCI^**a**^**	**ACPY^**b**^**	**Sum of times cited**	**IF^**c**^**
1	*Blood*	19	245	401	4,420	16.56
2	*Cancer Research*	9	165	114	1,492	8.38
3	*Science Translational Medicine*	9	507	456	4,565	17.16
4	*New England Journal of Medicine*	6	477	715	2,862	70.67
5	*Journal of Clinical Investigation*	6	324	194	1,946	12.28
6	*Nature Reviews Clinical Oncology*	6	237	178	1,425	34.11
7	*Nature Medicine*	6	236	236	1,418	30.64
8	*Cancer Immunology Research*	6	210	157	1,261	8.62
9	*Clinical Cancer Research*	6	211	126	1,266	8.91
10	*Journal of Clinical Oncology*	4	381	217	1,525	28.25

a *ACPI, average citations per item field (average number of times a record has been cited) (11)*.

b *ACPY, average citations per year (average number of times a record has been cited) (11)*.

c *Impact factor is based on Journal Citation Reports in June 2019*.

The authorial organizations and affiliations of the top 10 are summarized in Table 5. We found that the most-cited publications were authored in the University of Pennsylvania (*n* = 34), followed by the University of Texas (*n* = 15). However, the difference in citation frequency between the two was more than double. These are then proceeded by the Memorial Sloan Kettering Cancer Center (*n* = 15), University of Washington (*n* = 12), and the Fred Hutchinson Cancer Center (*n* = 11; Table 5). The University of Pennsylvania is the top-cited publication institution, along with the most-cited author (C. H. June; *n* = 29). Subsequently, authors B. L. Levine (University of Pennsylvania) and S. R. Riddell (Fred Hutchinson Cancer Research Center) published 12 articles each.

**Table 5:** Status of institutions/authors (affiliations) organizations that top 100 cited publications related to chimeric antigen receptor T-cell (CAR-T) from 2009 to 2019.

**Rank**	**Institutions**	**Number of publications**	**Authors (affiliation)**	**Number of publications**	**ACPI^**a**^**	**Sum of times cited**
1	University of Pennsylvania (UPENN)	34	Carl H. June (UPENN)	29	293	8,231
2	University of Texas MD Anderson (UTMD) Cancer Center	15	Bruce L. Levine (UPENN)	12	397	4,768
3	Memorial Sloan Kettering Cancer Center (MSKCC)	15	Stanley R. Riddell (FHCC)	10	257	2,575
4	University of Washington (UW)	12	Michel Sadelain (MSKCC)	10	346	3,465
5	National Cancer Institute (NCI)	12	John Scholler (UPENN)	9	199	1,592
6	Fred Hutchinson Cancer Center (FHCC)	11	Gianpietro Dotti (UT MD)	8	306	2,452
7	Baylor College of Medicine (BCM)	8	Michael C. V. Jensen (SCRI)	8	270	2,162
8	Children's Hospital of Philadelphia (CHOP)	7	Steven M. Albelda (UPENN)	8	208	1,669
9	Seattle Children's Research Institute	7	Stephan A. Grupp (CHOP)	7	520	3,640
10	National Institutes of Health (NIH)	6	Renier J. Brentjens (MSKCC)	7	261	1,831

a *ACPI, average citations per item field (average number of times a record has been cited) (11)*.

**Figure 3:**
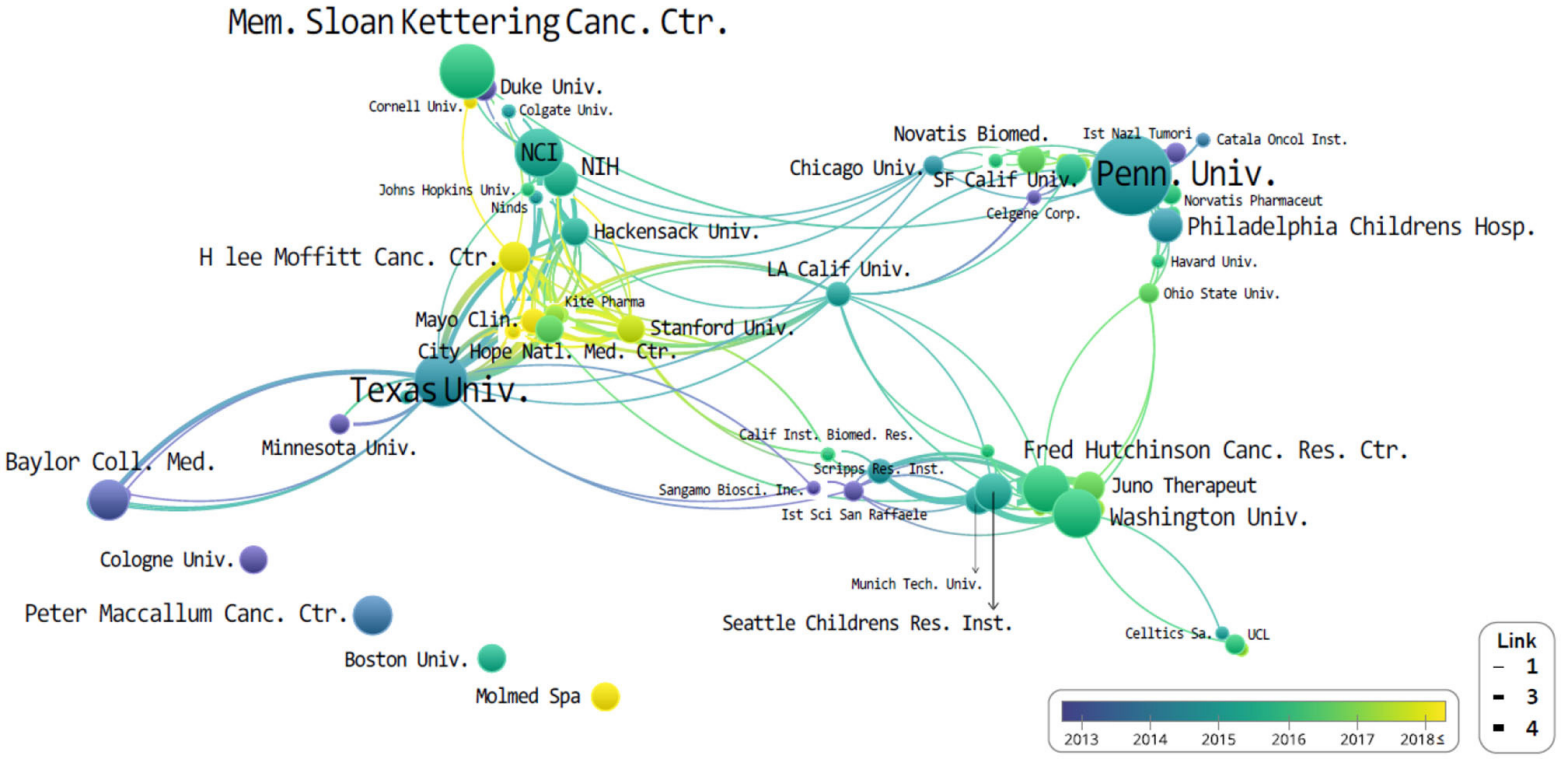
The network of institutions that co-published related to chimeric antigen receptor T-cell (CAR-T) from 2008 to 2019.

All of the organizations and authors of the top 10 most-cited publications were all from the USA. Figure 3 then illustrates the overall institution network graph. The network is centered around the University of Pennsylvania, Memorial Sloan Kettering Cancer Center, and University of Texas. Novartis is located close to the University of Pennsylvania and Kite Pharma to the University of Texas. On the other hand, the Fred Hutchinson Cancer Research Center collaborated primarily with Washington University, Juno Therapeutics, and the Seattle Children Research Institution. Looking at the co-authors. H. Lee Moffitt Cancer Center and Research Institute has published the most recent cited articles than both the University of Pennsylvania and MD Anderson. Nevertheless, no relationship is expressed in thick, or it is located as an independent organization.

The University of Pennsylvania is the top-cited publication, along with the most-cited author (C. H. June; *n* = 29). Subsequently, B. L. Levine (University of Pennsylvania) and S. R. Riddell (Fred Hutchinson Cancer Research Center) published 12 and 11 articles each, from the top 100 most-cited papers. Also notable was that the organizations and authors of the top 10 most-cited papers were all from the USA. L. A. Johnson connected between the J. N. Kochenderfer and C. H. June groups as a bridge role. In addition, M. Sadelain, G. Dotti, and S. R. Riddell have no clear connection bridge. It seems that each study has been conducted in small groups separately.

Finally, keywords from the most-cited 100 publications were extracted using “Keywords Plus” function from WoSCC. “KeyWords Plus are words or phrases that frequently appear in the titles of an article's references, but do not appear in the title of the article itself. Based upon a special algorithm that is unique to Clarivate Analytics databases, KeyWords Plus enhances the power of cited-reference searching by searching across disciplines for all the articles that have cited references in common” (11). We analyzed natural languages such as “treatment,” “Cell,” and “CAR” through independent reviewers by classifying them into the meaningless words. The most frequently used keywords were “adoptive immunotherapy” (*n* = 47), “lymphocytes” (n = 27), and “antitumor activity” (*n* = 22; Table 6).

**Table 6:** Frequency of keyword that top 100 cited publications related to chimeric antigen receptor T-cell (CAR-T) from 2008 to 2019.

**Keyword**	**Frequency**	**Keyword**	**Frequency**
Adoptive immunotherapy	47	Malignancy	10
Lymphocytes	27	Activation	10
Antitumor activity	22	Phase-I	10
Persistence	22	Sustained remissions	9
B-cell	16	PD-1	9
Acute lymphoblastic-leukemia	14	Remissions	8
Leukemia	11	Cytokine release syndrome	8
*In*-*vivo*	11	Adverse event	8
CD28	11	CD19	7
Transplantation	11	Non-Hodgkin-lymphoma	7

The density of the keywords is also accounted for and determined by their frequency of appearance (**Figure 5**). Higher-density keywords are represented in yellow, and lower densities are represented in blue; shorter distances between keyword nodes indicate frequent expression as co-occurring keywords.

## Discussion

We extracted the top 100 cited articles in the CAR-T field from the WoSCC database to analyze the field's network characteristics. In this study, we tried to visually express the research trends and mainstream structure through the network mapping of the simultaneous exposure of countries, funding bodies, researchers' affiliations and organizations, and keywords. Our network analysis found the USA and Germany possessed the most nodes, followed by Italy, Canada, and China. While an overwhelming amount of studies have been conducted in the USA, there appears to be an exchange with the vast majority of other countries (Figure 2).

**Figure 4:**
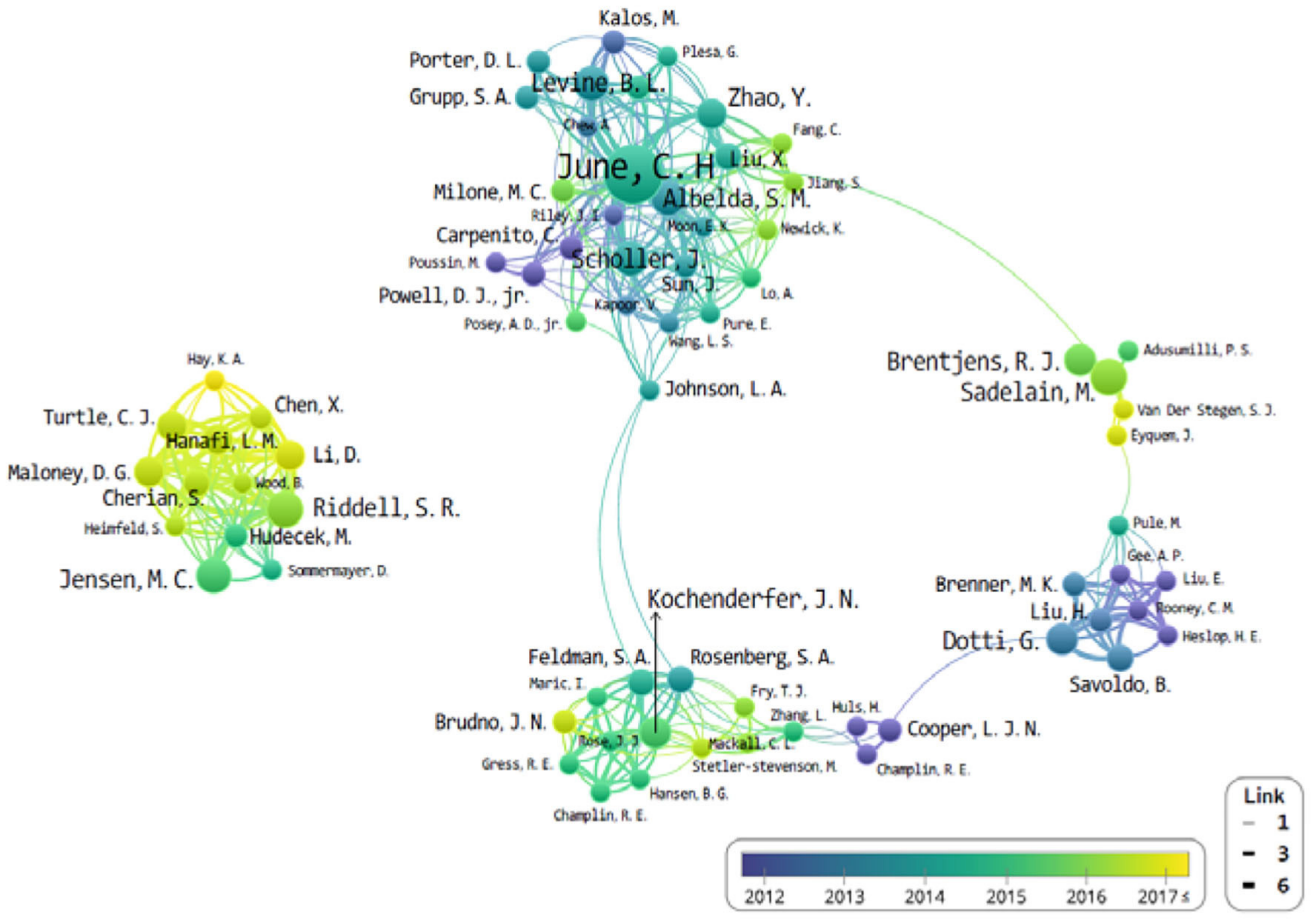
The network of authors that co-published related to chimeric antigen receptor T-cell (CAR-T) from 2008 to June 2019.

**Figure 5:**
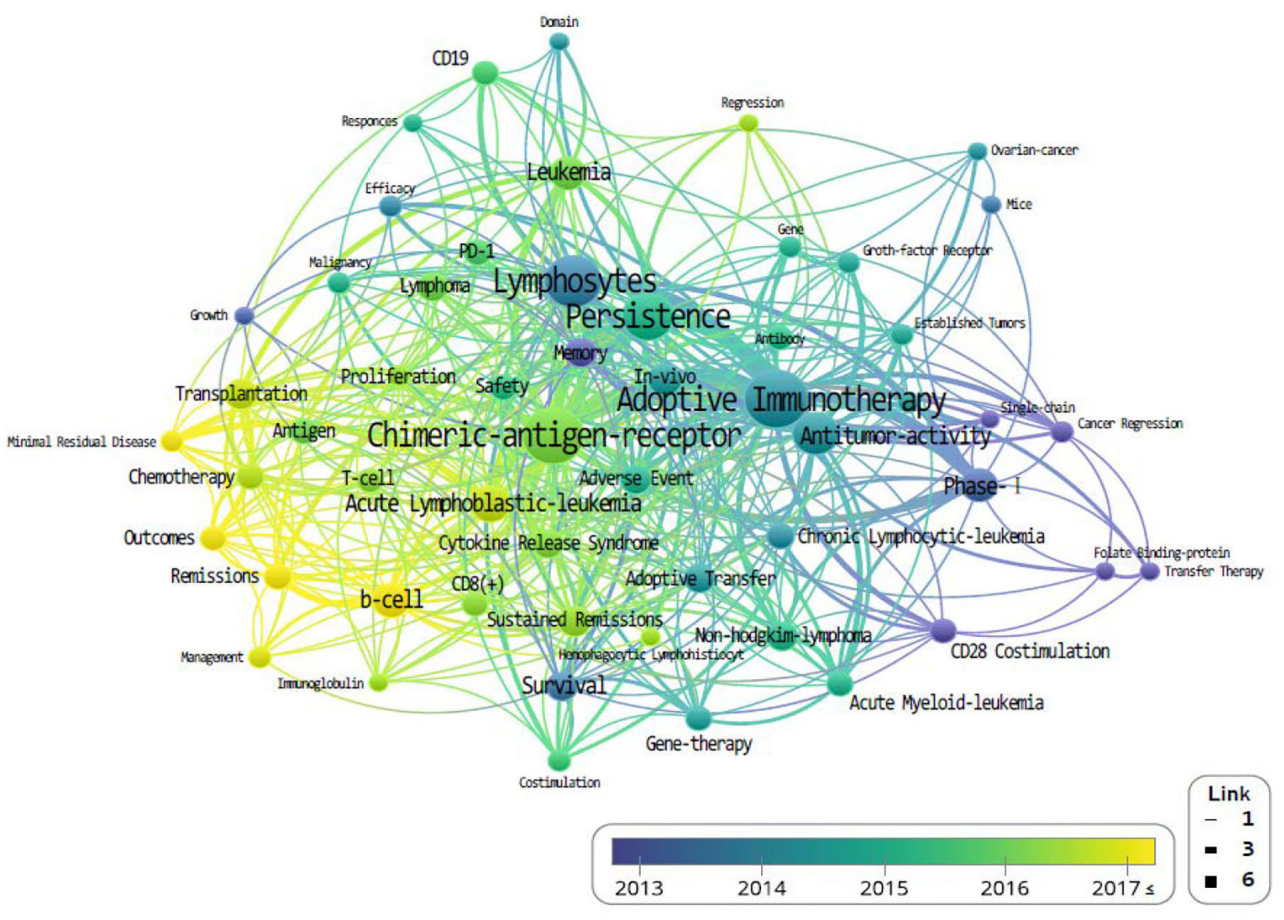
The co-occurrence density of keyword that co-published related to chimeric antigen receptor T-cell (CAR-T) from 1999 to June 2019.

The study of CAR-Ts is led mainly by three institutions: first is the University of Pennsylvania, then there is University of Texas MD Anderson (UTMD) Cancer Center, and Memorial Sloan Kettering Cancer Center (MSKCC) (Figure 3). When looking at the authorial connection and citation relationships, there seems to be a network centered around the works of C. H. June and B. L. Levine, with M. Sadelain acting as a bridging node. Another cluster of nodes centers around S. R. Riddell, and one around G. Dotti, but neither has a bridging node between them (Figure 4). This may imply that each group is being led by their respective institution directing the research and that there is minimal to no research network cooperation in the researches listed in ClinicalTrials.gov (7). Finally, the most used keyword is “adoptive immunotherapy,” which lies at the center, while other keywords like “lymphocytes,” “antitumor activity,” “persistence,” and “B-cell” were connected through multi-frequency simultaneous exposures (Figure 5).

In our determination of effect and value, we only consider the citation frequency. As such, we also only consider the number of co-authorships, regardless of an author's actual achievements and contributions. Future studies may consider the use of high quality databases that considers both qualitative and quantitative evidence for their objective analysis. Should consideration be placed on a study's phase and design, and the author's achievement and contribution for their analytical database, more robust results may be gained. Even now, there may be studies in press or in the process of completion that can contribute to the development of the field in spite of their low citation frequency.

CAR-T is a highly technological and high-cost method of cancer therapy; and the top funding bodies, journals, organizations, and authors were based or from the USA. The USA is objectively leading the development and research of the CAR-T field that is growing at an incredibly rapid rate (17–19). However, these advancements are not limited only to those dealing with CAR-T research and general cost-intensive treatments but also with overall immuno-chemotherapy research (20). In addition, CAR-T is a highly advanced next-generation treatment that has been studied in various fields such as lymphoma, solid cancer, rheumatoid arthritis, and autoimmune treatment. However, we have to consider the side effects of all treatments. Multiple cardiovascular adverse events are frequently stated after CAR-T therapy associated with mortality (21).

## Conclusion

Using the 100 most-cited papers in the CAR-T field, we attempted to provide insight into the direction of the scientific growth and core study areas and trends, and opportunity to understand information on the main network structure of those studies. What we observed was that CAR-T engineering is a developing technology- and cost-intensive form of immunotherapy, with most of its studies funded and led by US-based institutions and researchers.

## Data Availability Statement

The raw data supporting the conclusions of this article will be made available by the authors, without undue reservation.

## Author Contributions

All authors were involved in the design of the study. JK was responsible for critically reviewed, approved by all authors, and supervising. SK was responsible for data curation and visualization and contributed to data interpretation and critically reviewed all manuscript terms. BS originally draft writing, critically reviewed all manuscript version, and led this project. All authors agreed upon the final version of the manuscript.

## Conflict of Interest

The authors declare that the research was conducted in the absence of any commercial or financial relationships that could be construed as a potential conflict of interest.

## References

[1] Lim WA June CH. The principles of engineering immune cells to treat cancer. Cell. (2017) 168:724–40. 10.1016/j.cell.2017.01.016 28187291 PMC5553442

[2] Jackson HJ Rafiq S Brentjens RJ. Driving CAR T-cells forward. Nat Rev Clin Oncol. (2016) 13:370–83. 10.1038/nrclinonc.2016.36 27000958 PMC5529102

[3] Park JH Brentjens RJ. Adoptive immunotherapy for B-cell malignancies with autologous chimeric antigen receptor modified tumor targeted T cells. Discov Med. (2010) 9:277–88. 20423671 PMC4697441

[4] National Cancer Institute. Available online at: https://www.cancer.gov/about-cancer/treatment/research/car-t-cells

[5] Strohl WR Naso M. Bispecific T-cell redirection versus chimeric antigen receptor (CAR)-T cells as approaches to kill cancer cells. Antibodies. (2019) 8:41. 10.3390/antib8030041 31544847 PMC6784091

[6] Newick K O'Brien S Moon E Albelda SM. CAR T cell therapy for solid tumors. Annu Rev Med. (2017) 68:139–52. 10.1146/annurev-med-062315-120245 27860544

[7] Clinicaltrials.gov information. Available online at: https://clinicaltrials.gov/ct2/results?cond=CAR-T&Search=Apply&age_v=&gndr=&type=&rslt= (accessed July 1, 2019).

[8] Stolyarova E Beduleva L Menshikov I Sidorov A Khramova T. T lymphocyte dependence of the immune response to immunosuppressive neoantigen-exposing Fc fragments of IgG. J Biol Regul Homeost Agents. (2019) 33:869–76. 31186080

[9] Martinez M Moon EK. CAR T cells for solid tumors: new strategies for finding, infiltrating, and surviving in the tumor microenvironment. Front Immunol. (2019) 10:128. 10.3389/fimmu.2019.00128 30804938 PMC6370640

[10] McHugh J. CAR T cells drive out B cells in SLE. Nat Rev Rheumatol. (2019) 15:249. 10.1038/s41584-019-0214-x 30948844

[11] WoSCC detailed information. Available online at: https://images.webofknowledge.com/images/help/WOS/hp_citation_report.html (accessed July 1, 2019).

[12] Brandt JS Downing AC Howard DL Kofinas JD Chasen ST. Citation classics in obstetrics and gynecology: the 100 most frequently cited journal articles in the last 50 years. Am J Obstet Gynecol. (2010) 203:355.e1–7. 10.1016/j.ajog.2010.07.025 20875501

[13] Ponce FA Lozano AM. Highly cited works in neurosurgery. Part I: the 100 top-cited papers in neurosurgical journals. J Neurosurg. (2010) 112:223–32. 10.3171/2009.12.JNS091599 20078192

[14] Van Noorden R Maher B Nuzzo R. The top 100 papers. Nature. (2014) 514:550–3. 10.1038/514550a 25355343

[15] Eck V Jan N Waltman L. VOSviewer Manual. Leiden: Univeristeit Leiden (2013).

[16] Hartmann J Schüßler-Lenz M Bondanza A Buchholz CJ. Clinical development of CAR T cells-challenges and opportunities in translating innovative treatment concepts. EMBO Mol Med. (2017) 9:1183–97. 10.15252/emmm.201607485 28765140 PMC5582407

[17] Hoos A. Development of immuno-oncology drugs-from CTLA4 to PD1 to the next generations. Nature Rev Drug Discov. (2016) 15:235–47. 10.1038/nrd.2015.35 26965203

[18] Shi H Sun M Liu L Wang Z. Chimeric antigen receptor for adoptive immunotherapy of cancer: latest research and future prospects. Mol Cancer. (2014) 13:219. 10.1186/1476-4598-13-219 25241075 PMC4177696

[19] Tang J Shalabi A Hubbard-Lucey VM. Comprehensive analysis of the clinical immuno-oncology landscape. Ann Oncol. (2017) 29:84–91. 10.1093/annonc/mdx755 29228097

[20] Jürgens B Clarke NS. Evolution of CAR T-cell immunotherapy in terms of patenting activity. Nat Biotechnol. (2019) 37:370–5. 10.1038/s41587-019-0083-5 30940940

[21] Addison D Ghosh A Roddie C de Lima M Al-Kindi S Oliveira G. Cardiovascular events associated with CAR-T therapy. J Am Coll Cardiol. (2020) 75(Suppl 1). 10.1016/S0735-1097(20)30959-1

